# Mouse Model of Neurological Complications Resulting from Encephalitic Alphavirus Infection

**DOI:** 10.3389/fmicb.2017.00188

**Published:** 2017-02-07

**Authors:** Shannon E. Ronca, Jeanon Smith, Takaaki Koma, Magda M. Miller, Nadezhda Yun, Kelly T. Dineley, Slobodan Paessler

**Affiliations:** ^1^Department of Pathology, University of Texas Medical Branch, GalvestonTX, USA; ^2^Department of Preventive Medicine and Community Health, University of Texas Medical Branch, GalvestonTX, USA; ^3^Department of Neurology, Center for Addiction Research, University of Texas Medical Branch, GalvestonTX, USA; ^4^Galveston National Lab, Institute for Human Infections and Immunity, University of Texas Medical Branch, GalvestonTX, USA

**Keywords:** alphavirus, VEEV, TC83, sequelae, neurological complications

## Abstract

Long-term neurological complications, termed sequelae, can result from viral encephalitis, which are not well understood. In human survivors, alphavirus encephalitis can cause severe neurobehavioral changes, in the most extreme cases, a schizophrenic-like syndrome. In the present study, we aimed to adapt an animal model of alphavirus infection survival to study the development of these long-term neurological complications. Upon low-dose infection of wild-type C57B/6 mice, asymptomatic and symptomatic groups were established and compared to mock-infected mice to measure general health and baseline neurological function, including the acoustic startle response and prepulse inhibition paradigm. Prepulse inhibition is a robust operational measure of sensorimotor gating, a fundamental form of information processing. Deficits in prepulse inhibition manifest as the inability to filter out extraneous sensory stimuli. Sensory gating is disrupted in schizophrenia and other mental disorders, as well as neurodegenerative diseases. Symptomatic mice developed deficits in prepulse inhibition that lasted through 6 months post infection; these deficits were absent in asymptomatic or mock-infected groups. Accompanying prepulse inhibition deficits, symptomatic animals exhibited thalamus damage as visualized with H&E staining, as well as increased GFAP expression in the posterior complex of the thalamus and dentate gyrus of the hippocampus. These histological changes and increased GFAP expression were absent in the asymptomatic and mock-infected animals, indicating that glial scarring could have contributed to the prepulse inhibition phenotype observed in the symptomatic animals. This model provides a tool to test mechanisms of and treatments for the neurological sequelae of viral encephalitis and begins to delineate potential explanations for the development of such sequelae post infection.

## Introduction

Neurological sequelae are a widespread and understudied consequence of virus encephalitis that can have profound influence on the survivor’s quality of life. Neurological sequelae are those complications involving damage to the central nervous system that results in cognitive, sensory, or motor deficits that may also manifest as emotional instability and even seizures, in the most severe cases. Sequelae affect persons in all age groups and levels of immunocompetence. Neurological complications resulting from viral infections have recently become the focus of much media attention. For example, Zika infection is apparently associated with developmental microcephaly and Guillain-Barré syndrome (Wu et al., 2016), Chikungunya infection with arthritis (Rampal et al., 2007; Bhavana et al., 2008; Chusri et al., 2011), Ebola virus infection with sensory impairment and behavioral changes, and Lassa virus infection with hearing loss (Cummins et al., 1990; Okokhere et al., 2009; Ibekwe et al., 2011).

Venezuelan equine encephalitis virus (VEEV), eastern equine encephalitis virus (EEEV), and western equine encephalitis virus (WEEV) are the three most common encephalitic alphaviruses associated with neurological sequelae in the Americas (Mulder et al., 1951; Finley and Chapman, 1953; Fulton and Burton, 1953; Palmer and Finley, 1956; Hanson, 1957; Herzon et al., 1957; Cohn and Kuida, 1962; Earnest et al., 1971; Bowen et al., 1976; White et al., 1983; Deaton et al., 1986; Carrera et al., 2013; Silverman et al., 2013). Sequelae of alphavirus infection include seizures, photophobia, intellectual disability, emotional instability, behavioral changes, neuromuscular weakness, and paralysis and the incidence of these symptoms can occur in up to 75% of surviving patients (Mulder et al., 1951; Finley and Chapman, 1953; Fulton and Burton, 1953; Palmer and Finley, 1956; Hanson, 1957; Herzon et al., 1957; Cohn and Kuida, 1962; Earnest et al., 1971; Bowen et al., 1976; White et al., 1983; Deaton et al., 1986; Carrera et al., 2013; Silverman et al., 2013). When evaluating extreme outcomes, one unfortunate patient was misdiagnosed and treated for schizophrenia after recovering from an undetected WEEV infection. Eighteen months passed before doctors were able to correctly link the patient’s symptoms as neurological sequelae of encephalitis (Deaton et al., 1986). This is not the only example of severe behavioral symptoms following viral encephalitis. Studies in two Canadian psychiatric hospitals observed that 32 patients had antibodies against WEEV or a related encephalitic virus (Fulton and Burton, 1953), leading the authors to suggest that viral infection may have contributed to the patients neurological disease.

The economic burden resulting from alphavirus (EEEV) neurological sequelae can exceed $3 million per patient per year (1995 dollars) (17). This estimate did not include the costs of initial hospitalization, estimated to be $21,000 per patient per week, nor the cost of institutionalization for patients that do not have family members to aid in long-term convalescence (Villari et al., 1995). Thus, relevant animal models with appropriate face validity for the neurological sequelae resulting from alphavirus encephalitis are crucial for accurate diagnosis and effective treatments; not to mention enhanced quality of life and relief from the economic burden associated with surviving these infections.

We initiated validation of a mouse model for VEEV neurological sequelae by infecting C56BL/6 with TC-83, an attenuated VEEV strain (McKinney et al., 1963). By using TC-83, we were able to adapt biosafety level (BSL)-1 neurobehavioral techniques in a BSL-2 setting. This is valuable, as studies will need to be translated to BSL-3 to study the wild-type strains of VEEV and the other encephalitic alphaviruses. We evaluated baseline neurological function in mice using the SHIRPA, acoustic startle reflex (ASR), and prepulse inhibition paradigm (PPI). SHIRPA is a semi-quantitative neurological assessment of general health, neurological reflexes, and baseline behavior (Rogers et al., 1997, 2001). ASR evaluates an animal’s ability to perceive and respond to acoustic startle stimuli while PPI assesses sensorimotor gating of the ASR stimuli (Koch, 1999); thus, allowing the evaluation of a multi-synaptic reflexive brain circuit in hearing-competent animals (Koch and Schnitzler, 1997; Koch, 1999; Fendt et al., 2001; Crawley, 2007). Deficits in PPI is related to a variety of psychobehavioral diseases, such as schizophrenia (Braff et al., 1978, 2001a,b; Davis et al., 1982; Grillon et al., 1992; Koch, 1999), attention deficit disorder/attention deficit hyperactivity disorder (Castellanos et al., 1996; Kohl et al., 2013), anxiety (Ludewig S. et al., 2002; Kohl et al., 2013), depression (Kohl et al., 2013), and Alzheimer’s disease (Salem et al., 2011; Wang et al., 2012; Koppel et al., 2014). Rodent animal models for ASR and PPI deficits have been developed and successfully used to model the sensorimotor gating deficits of these diseases. However, neurobehavioral testing has yet to be used to evaluate neurological sequelae due to arboviral infection in mice (Fendt and Koch, 1999; Schauz and Koch, 1999; Swerdlow et al., 1999, 2001; Geyer et al., 2001, 2002). In addition to these neurobehavioral tests, we will use histological staining of brain cell populations to evaluate neurodegeneration using NeuN as a marker, an antibody specific to mature neurons. Activation of astrocytes can lead to neuroinflammation and cell damage. This will be evaluated using glial fibrillary acidic protein (GFAP) as a marker. Evaluating astrocyte activation and neurodegeneration of animals with different symptom presentations provides initial insight into mechanisms of damage. Thus, we describe a novel approach to study neurological sequelae resulting from alphavirus encephalitis

## Materials and Methods

### Cells, Viruses, and Biosafety

Vero cells (American Tissue Culture Collection CRL-1586) were maintained in Dulbecco’s modified Eagle’s medium supplemented with 10% fetal calf serum, 1% penicillin-streptomycin, and L-glutamine. Viral working stocks were produced in Vero cells. All work with infectious virus was performed at UTMB BSL-2 or 3 in accordance with institutional health and safety guidelines.

### Animal Experiments

Six to eight-week-old female wild-type C56BL/6 were purchased from Jackson Laboratories (Bar Harbor, ME, USA). All animals were housed in a pathogen-free environment. All virus infections were performed in the ABSL2 or ABSL3 facility in the Galveston National Laboratory (GNL), UTMB. All animal studies were reviewed and approved by the Institutional Animal Care and Use Committee at UTMB and were carried out in accordance with the National Institute of Health guidelines. Animals were anesthetized using an isoflurane precisions variable-bypass vaporizer prior to intranasal virus inoculation with 10^3^ PFU TC-83 diluted in PBS (*N* = 10, performed as two separate experiments of *N* = 5) or PBS as a mock infection (*N* = 4). Standardized recording of death and disease symptoms was performed using the following definitions: encephalitis, ruﬄed fur, hunched back, reduced activity, development of discoordination, ataxia, or transient seizures (with the ability to drink and feed); paralysis; and hind limb (hemiplegic) or quadriplegic paralysis (with the inability to reach the feeder or water bottle). Body weight was measured throughout the course of study. Seroconversion was determined by plaque reduction neutralization test (PRNT) at 6 months post infection (m.p.i.).

### General Health Assessment

A modified SHIRPA screen was used to analyze the general health of each animal pre- and post-infection. Semi-quantitative measures were performed to evaluate body tone, trunk curl, righting, reach touch, whisker response, ear twitch, palpebral reflex, forelimb place, and grip time. Semi-quantitative measurements were ranked on a sale of 0 to 3, with 0 being the lowest possible, 1 equating to less than normal, 2 equating to normal, and 3 equating to hyperactive (**Table 1**). The body tone test was done by using two fingers to depress along the spine of the animal and record the animal’s response. Trunk curl was done by holding the animal by the base of the tail above the ground and observing the angle measured vertically against a sheet of marked paper to which the animal curls. A normal animal should be able to curl to at least 90°. The righting reflex was determined by flipping the animal on its back in the palm of a hand and evaluating its ability to bring itself back on all four paws. Reach touch was evaluated by holding the animal from the base of the tail above a surface and slowly lowering it towards the ground. A normal animal will reach its front paws towards the surface as it is lowered, indicating intact vision. The whisker response and ear twitch tests were performed by lightly flicking the whisker or ear with an elongated Q-tip from behind and observing the animal’s response. The palpebral reflex test was done by slowing approaching the eye with a cotton-tipped swab and observing when/if the animal responds by blinking. The forelimb place was done with the animal sitting with all four paws on a flat surface. Each paw was moved to the side using forceps and the animal’s response was observed. It is expected that the animal will return its forelimb close to its original position and it is abnormal for the animal not to fix the placement of its paw. Grip time was determined by placing an animal on a wire cage top and turning the cage top upside down. Normal animals should be able to hold on to the cage for at least 60 s, so this test was concluded once the animal’s grip time exceeded 60 s. The above described tests were done as previously described (Rogers et al., 1997; Crawley, 2007). The response to each was rated on a scale of 0 to 3, with 0 indicating no response, 1 indicating a less than normal response, 2 indicating a normal response, and 3 indicating an exaggerated response. We established baseline SHIRPA 1 week prior to infection. Post-infection measurements were evaluated beginning 1 month post infection through the end of the study at 6 m.p.i.

**Table 1:** SHIRPA scoring.

	0	1	2 (Normal)	3
Body tone (muscular, agitation)	Flaccid, no reaction	Depression to floor	Slight resistance to touch	Hunches back to resist depression, irritable
Trunk curl (muscular)	No response or hindlimb clasping	Curls < 90°	Curls ≥ 90°	Climbs up tail
Righting (muscular)	Does not right	Struggles to right	Rights itself	Hyperactive response
Forelimb place (muscular)	Leg stays where placed	Slow or incomplete return to position	Promptly returns to position	Hyperactive response, does not allow adjustment
Palpebral Reflex (visual)	No response	Slow blink	Quick blink	Continues blinking upon removal of stimulus
Reach touch (visual)	Does not reach, clasps hindlimbs	Does not reach until whiskers touch surface	Reaches as approach ground before whiskers touch surface	Hyperactive, tail climbing

### Acoustic Startle Reflex (ASR) and Prepulse Inhibition (PPI)

Acoustic Startle Reflex (ASR) was used to measure reactions to startle-eliciting sounds pre- and post-TC-83 infection in WT C56BL/6 mice. Baseline ASR was established 1 week prior to infection and 1, 3, and 6 m.p.i. Each animal was placed in a tube restraint large enough for the animal to turn around in then placed in the SR-Lab startle response system sound attenuating cabinet (San Diego Instruments). The SR-Lab startle response system recorded the amplitude of each animal’s movement in response to acoustic startle stimuli across a 100 ms window following stimulus onset. Experimental parameters and stimuli presentations were controlled with SR-Lab software. Prior to the onset of the experiment, each animal was subjected to a 3–5-min acclimation period in the cabinet. A startle stimulus of 120 d B was presented as 20 ms bursts in the presence of a 65 dB background noise. The control stimulus consisted of 20 ms presentations of the 65 dB background noise. The acoustic stimuli were presented in a randomized order with a total of 10 trials for each option. The inter-trial interval varied between 10 and 20 s. The amplitude of the animal’s movement was measured in mV.

Prepulse inhibition paradigm (PPI) was evaluated 1 week pre-infection and 1, 3, and 6 m.p.i. for all animals. Animals were acclimated to the chamber as described above for ASR. Within 100 ms prior to the 120 dB startle stimulus, prepulses at 6, 12, and 18 dB above the 65 dB background were presented. No prepulse prior to the 120 dB startle served as baseline. Prepulse stimuli were presented in a randomized order with a total of 10 trials for each option. The inter-trial interval varied between 10 and 20 s. The amplitude of the animals’ movement was measured at each prepulse level and the percent inhibition was calculated using the following equation: 100[(Startle Amplitude – PPI Startle Amplitutde)/Startle Amplitude].

### Brain Histology

At the conclusion of the study (6 m.p.i), mice were killed with carbon dioxide. Whole brains (Mock *N* = 4, Asymptomatic *N* = 7, Symptomatic *N* = 3) were removed, fixed in 10% buffered formalin (Sigma–Aldrich, HT501128-4L), then embedded in paraffin by the University of Texas Medical Branch Histology Core (directed by Dr. Marjan Afrouzian). Five micrometer sagittal slices were cut on a Leica RM2125 microtome then stained with hematoxylin and eosin or with primary and secondary antibodies for immunofluorescence microscopy. Primary antibodies employed were: NeuN (Cell Signaling D3S3I Rabbit mAB, 1:500), and GFAP (Aves Labs, Inc. Chicken mAB Catalog#GFAP, 1:2000). Secondary antibodies used were: goat anti-rabbit IgG (DyLight 488ThermoFisher Catalog#35552, 1:250) and goat anti-chicken IgY (DyLight 594 ThermoFisher Catalog#SA5-100072, 1:250). Sections were coverslipped with DAPI-containing mounting media (DAPI Fluoromount-G, SouthernBioTech). GFAP-positive cells were quantified by counting the number of GFAP-positive cells in the 20x field of view centered either on the posterior complex of the thalamus (PO) or dentate gyrus (DG), then counting the number of NeuN-positive cells in that field. The field of view for each animal was determined and replicated using a brain atlas (Paxinos and Franklin, 2004) such that comparisons between animals occurred using three slices, each corresponding to 0.6–0.85 mm lateral sections as determined through the use of the atlas. The ratio of GFAP:NeuN positive cells were determined for three consecutive slices for animals from each group, again with the slices falling within 0.6–0.85 mm lateral sections. DAPI-staining was used to normalize the total number of cells within a field of view.

### Statistical Analyses

Each group’s weight (symptomatic, asymptomatic, and mock) was compared using one-way ANOVA. A within group paired t-test was used to compare baseline ASR amplitudes and percent inhibition in the PPI test to post-infection values, according to previously published studies (Yee et al., 2005). A p-value of less than 0.05 was considered statistically significant for all tests. The ratios of GFAP:NeuN-positive cells were compared between each group (mock, asymptomatic, symptomatic) using one-way ANOVA.

## Results

### General Health Assessment

**FIGURE 1:**
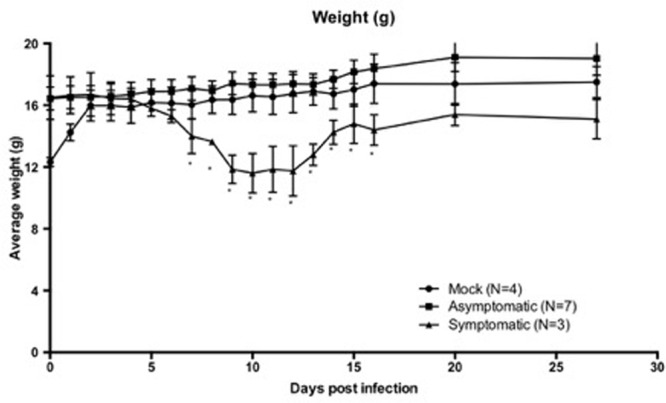
**Six-to-eight-week-old wild-type C57BL/6 mice infected with TC-83 or mock infected with PBS were monitored throughout the course of study.** Symptomatic (S, *N* = 3) animals developed weight loss consistent with infection around D5 and did not begin regaining weight until D11. Asymptomatic (A, N = 7) and mock (M, *N* = 4) animals did not develop weight loss or show other signs of infection. S animals did not reach weights comparable to the A or M animals until D20. Weights are expressed with grams with the SD for each group. Asterisks indicate significantly lower weights (*p* < 0.05) in the symptomatic groups when compared to both asymptomatic and mock groups. When compared using a One-Way ANOVA, weights were not significantly different between mock or asymptomatic groups post-infection.

WT C56BL/6 mice were infected with 10^3^ PFU TC-83 (*N* = 10) or mock infected with an equal volume of PBS (*N* = 4). Infected animals were divided into symptomatic (*N* = 3) and asymptomatic (*N* = 7) animals based on the development of weight loss and disease symptoms. Symptomatic animals displayed disease symptoms of ruﬄed fur, hunched back, and reduced activity recorded within 14 d.p.i, while asymptomatic animals did not display these disease characteristics and maintained an overall healthy appearance post-infection within 14 d.p.i. All symptomatic animals developed weight loss consistent with disease by 5 d.p.i. and recovered weight by 11 d.p.i (**Figure 1**). However, these symptomatic mice had lower body weights at the time of infection than their asymptomatic cage mates. By 2 d.p.i., all animals had comparable weights. We are unable to determine if this affected the development of disease symptoms. As expected, no animals succumbed to challenge. Seroconversion, as measured by PRNT (data not shown), occurred in all symptomatic animals and 2 out of 7 asymptomatic animals. Asymptomatic animals that did not seroconvert were still considered infected due to the low dose infection used and the poor immunogenicity established for TC83 (Pittman et al., 1996).

All animals were evaluated for behavioral changes until 6 m.p.i., when the study was terminated. The SHIRPA (Rogers et al., 2001) general health and neurological screen was used to reveal discrete neuromuscular phenotypes in infected animals (**Table 2**). Baselines were established for each animal 1 week prior to infection and then were evaluated weekly beginning 1 m.p.i. until 6 m.p.i. Abnormalities were identified in righting reflex, grip latency, forelimb place, body tone, and trunk curl for infected animals but not animals in the mock group (**Table 2**). The only animals that sustained abnormalities in grip time, righting, and forelimb place from 1 m.p.i. to 6 m.p.i were those that displayed symptoms post-infection, as described above. However, both symptomatic and asymptomatic animals presented with abnormalities in body tone and trunk curl from 1 m.p.i. to 6.m.p.i. It is important to note that not all animals exhibited the same directionality of altered response to trunk curl and body tone; with 6 showing an attenuated response and 4 showing an exaggerated response.

**Table 2:** General health changes pre- and post- infection.

	Body Tone	Trunk Curl	Righting	Forelimb place	Grip Time
Pre-infection (1 week) Infected Mock	Normal/Abnormal 10/0 4/0	Normal/Abnormal 10/0 4/0	Normal/Abnormal 10/0 4/0	Normal/Abnormal 10/0 4/0	Normal/Abnormal 10/0 4/0
Post-infection (1 month) Infected Mock	Normal/Abnormal 0/10 4/0	Normal/Abnormal 0/10 4/0	Normal/Abnormal 9/1 4/0	Normal/Abnormal 8/2 4/0	Normal/Abnormal 8/2 4/0
Post-infection (6 month) Infected Mock	Normal/Abnormal 0/10 4/0	Normal/Abnormal 0/10 4/0	Normal/Abnormal 9/1 4/0	Normal/Abnormal 9/1 4/0	Normal/Abnormal 8/2 4/0

### Acoustic Startle and Prepulse Inhibition

**FIGURE 2:**
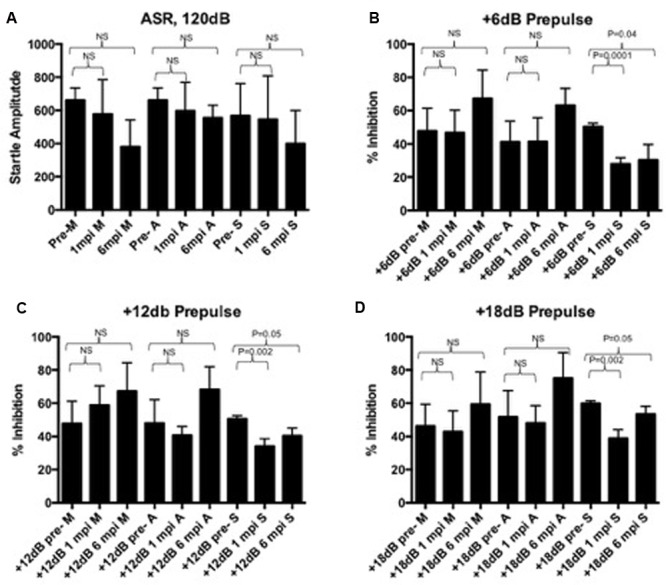
**(A)** The startle response is unaffected pre- and post-infection in mock, asymptomatic, and symptomatic groups. A slight statistically insignificant decrease over time is expected due to long-term habituation to the testing. At 6dB **(B)**, 12dB **(C)**, or 18dB **(D)** above background noise, all groups can reach up to 60% inhibition of the startle response. At 1 m.p.i and 6 m.p.i, this percent inhibition is significantly decreased in symptomatic animals, but not in mock or asymptomatic animals. Baseline and post-infection time points were compared using a paired *t*-test.

To determine the sensorimotor gating deficits associated with PPI, the baseline startle response and was established and we confirmed functional hearing prior to PPI evaluation (**Figure 2**). The startle response was statistically unchanged in any group when compared to its pre-infection baselines (**Figure 2A**). The startle response is expected to decrease slightly over time due to habituation to the procedure, and as expected, appears to decrease but was not a significant decrease (**Figure 2A**). However, a decrease in percent inhibition at three different prepulse intensities (+6, 12, and 18 dB above background) was statistically significant for animals in the symptomatic group at 1 m.p.i. (*p* = 0.04, *p* = 0.05, *p* = 0.05) that continued to be significant through 6 m.p.i. (*p* = 0.0001, *p* = 0.002, *p* = 0.002) (**Figures 2B–D**) when compared to baseline pre-infection measurements. This decrease was not detected in mock or asymptomatic animals at any of the prepulse intensities (**Figures 2B–D**). Thus, while asymptomatic mice exhibited muscle weakness and gait abnormalities as measured by SHIRPA, only symptomatic mice displayed CNS circuit abnormalities as measured by PPI.

### Histopathological Changes

**FIGURE 3:**
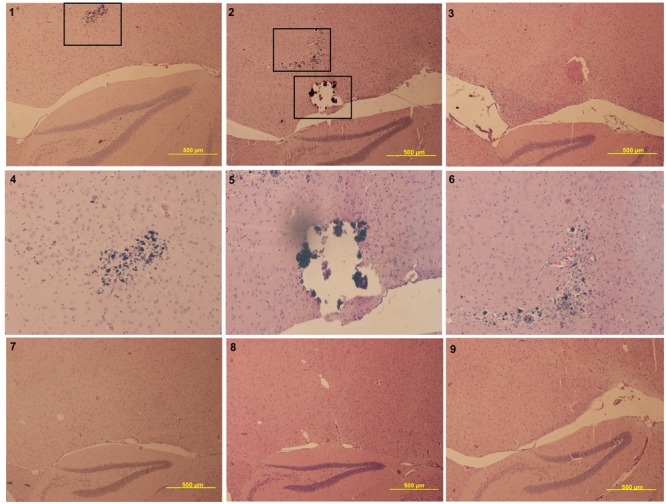
**Panels 1–3 are symptomatic animals (4x magnification lens), panel 4–6 represents zoom-in panels of the boxed damage of panels 1 and 2 (10× magnification lens), panel 7 represents a mock animal (4× magnification lens), and panels 8–9 represent asymptomatic animals (4× magnification lens).** All symptomatic animals had visible damage to the thalamus, such as the glial nodules (panels 1 and 2, higher magnification in panels 4 and 6) and calcification (panel 2, higher magnification in panel 5. This same damage is not seen in mock (panel 7) or asymptomatic (panels 8–9) animals.

Next, brains of symptomatic, asymptomatic, and mock-infected animals were compared. At the conclusion of the study (6 m.p.i), whole brains were collected from the animals and fixed in 10% buffered formalin. Five um sagittal brain slices were stained with hematoxylin and eosin to resolve general brain structure and potential damage. Symptomatic animals displayed damage to the thalamus, as indicated by enhanced hematoxylin deposition, with one animal displaying glial nodules and damage possibly due to calcification (**Figure 3**). Interestingly, this damage was not seen in mock or asymptomatic animals (**Figure 3**).

**FIGURE 4:**
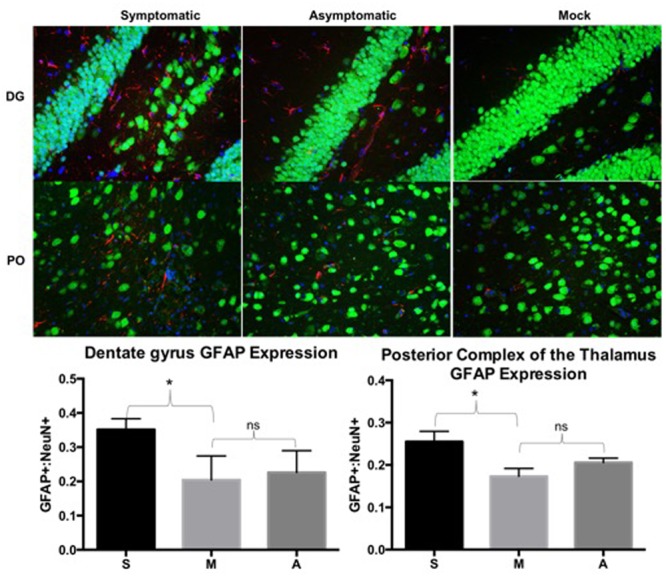
**Symptomatic animals had more GFAP expression than both asymptomatic and mock groups in the dentate gyrus (DG) and posterior complex of the thalamus (PO).** Asymptomatic animals had more GFAP expression than the mock animals, but less so than symptomatic animals. GFAP expression was determined by dividing counts of GFAP positive cells by NeuN positive cells in comparable fields of view. Representative images from each group are depicted. Green: NeuN, Red: GFAP, Blue: DAPI. ns: not significant, ^∗^*p* < 0.005. S: symptomatic; M: mock; A: asymptomatic.

Slices were next probed for NeuN to detect mature neurons, GFAP to detect activated astrocytes/microglia, and DAPI to identify cell nuclei. The ratio of GFAP-positive to NeuN-positive cells was determined for each field of view. Symptomatic, asymptomatic, and mock animals were compared by the ratio of GFAP:NeuN for at least 3 consecutive slices from each animal in comparable regions of the brain (i.e., thalamus, dentate gyrus, and superior and inferior colliculi). Increased GFAP:NeuN ratios were detected in symptomatic animals when compared to mock in the dentate gyrus (DG) (*p* = 0.0014) and posterior complex of the thalamus (PO) (*p* = 0.0049) (**Figure 4**). Although there seemed to be a trend of increased GFAP expression in asymptomatic animals compared to mock animals, this was not statistically significant in the DG or PO (**Figure 4**). There was no significant change in GFAP:NeuN ratios in the superior or inferior colliculi (data not shown).

## Discussion

This study describes the initial validation of a mouse model for neurological complications that can occur post-alphavirus infection. Validation of an animal model requires that the model have face validity, construct validity, and predictive validity. Face validity requires that the model mimic human symptoms, construct validity that the model replicate structural and neurochemical defects, and predictive validity ensures that the model reflects responses to treatment that are described in human use (Chadman et al., 2009; Jones et al., 2011). At this stage, this model meets the criteria described for face validity and the overt structural defects supports construct validity.

In this model, low-dose infection with TC-83 led to the development of encephalitis and survival for long-term study. For the first time, the detectable changes in general health, neurological reflexes, sensory function, and sensorimotor gating were observed and quantified in the context of alphavirus infection (**Figures 1** and **2**; **Table 2**). It was anticipated that the low-dose of infection would not lead to detectable infection in all animals, thus providing a way to evaluate sequelae of symptomatic and asymptomatic infections. All symptomatic animals seroconverted by the end of the study (6 m.p.i.), while only 2 of the 7 asymptomatic animals showed evidence of seroconversion at that time. Thus, these findings support the face validity of this model to mimic human alphavirus infection, as these infections can range from asymptomatic to encephalitis.

Infected animals had abnormalities in neuromuscular tests, which indicate that there may be damage to nerves that relate to the control of voluntary and reflex muscle movement. This is consistent with what is described in human disease and further supports the face validity of this model. All animals infected with TC-83 displayed abnormalities in their body tone and trunk curl. The body tone test, sometimes called touch escape, evaluates the animal’s physical response upon slight depression along the spine towards the head. Infected mice were irritable upon touch (*N* = 4), while others allowed depression to the floor and did not react to the physical pressure (*N* = 6). The trunk curl test also evaluates the mouse’s neuromuscular abilities by evaluating the approximate angle that the mouse can pull itself up when held by the base of the tail (Crawley, 2007). Only some symptomatic animals had abnormalities in grip time (less than 60 s) (*N* = 2), forelimb place (*N* = 1), and righting (*N* = 1) lasting through 6 m.pi. Abnormalities in these general reactivity tests could indicate a physical or muscle problem in animals with less than normal responses, potentially due to the descending projections of the motor tracts in the somatic or autonomic nervous systems. Alternatively, an exaggerated response could indicate irritability. In fact, the exaggerated response to body tone assessment has been indicated as a model for non-cognitive symptoms associated with dementia (Reisberg et al., 1987). This exaggerated response and associated irritability are interesting because case reports of alphavirus infections have described dementia as one possible sequelae, and the sequelae of alphaviruses are not all related to cognition. At this stage, the neuromuscular deficits detected in this model correlate with those that are described in alphavirus encephalitis survivors, as well as individuals infected with West Nile Virus (Mulder et al., 1951; Bruyn and Lennette, 1953; Palmer and Finley, 1956; Herzon et al., 1957; Earnest et al., 1971; Leon, 1975; Bowen et al., 1976; Patel et al., 2015).

Infection did not alter the basic startle response amplitude over the 6 months of observation, but symptomatic infection led to deficits in PPI (**Figure 2**). To understand what was observed in this study, one must understand the ASR and PPI pathways. Upon presentation of a startle stimulus, the soundwave travels through the cochlea to activate the appropriate hair cells that communicate acoustic information to the caudal pontine reticular nucleus, sending the signal for the motor response to the motor neurons (Fendt et al., 2001). When a prepulse is presented at the appropriate interval prior to the startle stimulus, the prepulse is processed via the default pathway, rerouting the startle stimulus through the inferior and superior colliculi from the cochlear nuclei, then to the pedunculopontine reticular nucleus before being re-routed to the caudal pontine reticular nucleus and motor neurons of the traditional acoustic pathway (Fendt et al., 2001). From the caudal pontine reticular nucleus, the signal for the motor reflex is sent through the spinal cord to elicit a motor response. This is done by transferring a signal to the spinal interneurons, which are responsible for creating the neural circuits that relay information between sensory and motor neurons, which then relay the signal to spinal motor neurons for the physical response (Yeomans and Frankland, 1995). There are many aspects of the startle response to evaluate to determine the cause of deficits in sensorimotor gating and the motor response. The observation that the startle response was unaltered over the course of the study, but the alterations in percent inhibition of symptomatic animals indicates impairment in sensorimotor gating, a reflexive process that blocks redundant stimuli and prevents an overload of information. This impairment is a phenotype associated with neurological disorders, including but not limited to schizophrenia, ADD/ADHD, Alzheimer’s, and Autism spectrum disorders (Grillon et al., 1992; Kumari et al., 1999; Braff et al., 2001b; Ludewig K. et al., 2002). Overall, the results observed regarding ASR and PPI measurements correlate with the kinds of sequelae expected from human cases.

The pathological lesions observed are relevant to the outcomes measured by the SHIRPA examination, ASR, and PPI. In this study, mice with PPI deficits exhibited damage in the thalamus, but not to other areas of the brain (**Figure 4**). The thalamus is situated between the cortex and the midbrain, acting as a relay between different subcortical areas and the cortex. Thus, it is involved in sensory perception, regulation of motor functions, regulation of reflexes from auditory and visual stimuli, and consciousness. The thalamus has been noted as a region of importance for neurological diseases including schizophrenia, depression, anxiety, Parkinson’s disease, and epilepsy. Rodent models of schizophrenia provide valuable information regarding changes in PPI, but this information can be applied to other neurological disorders. For example, disruption of the thalamic inputs have been described to play a role in sensorimotor gating deficits (Chun et al., 2014). Additionally, Klein and colleagues observed that deep brain stimulation of the dorsomedial thalamus normalized PPI deficits in two rat models (Klein et al., 2013) and Alsene and colleagues determined that infusion of norepinephrine into the dorsomedial thalamus disrupted PPI in mice (Alsene et al., 2011). Information regarding the functions of the thalamus and its role in rodent models of neurological disease resulting in disrupted PPI supports the structural basis for construct validity of the neurological disorders in the mouse model described here.

To further evaluate the construct validity of the structural damage in the context of virus infection, previous histopathology reports of encephalitic alphavirus infection in horses (Monlux and Luedke, 1973; Monath et al., 1981; Del Piero et al., 2001; Silva et al., 2011), guinea pigs (Roy et al., 2009), deer (Kiupel et al., 2013), and nonhuman primates (Nathanson et al., 1969) were examined. These reports identified virus in the thalamus and thalamic damage as a result of infection similar to what is described in this study. In fact, virus has been detected in the thalamus of lethal human cases (Nathanson et al., 1969; Luby et al., 1971). However, due to the lethality of the virus, these previous reports were only able to evaluate the results of acute infection leading to death and did not look at the long-term effects of infection in this region and in the host, such as PPI, ASR, and other behavioral outcomes. Further investigations are required to delineate the role of thalamus damage to these complications of infection and evaluate the predictive validity of the model using methods to stimulate the thalamus and reverse PPI deficits.

Additional phenotypic measures to provide construct validity were evaluated. This was accomplished through GFAP and NeuN staining. GFAP is an intermediate filament protein expressed by astrocytes in the CNS. It’s direct role in neurological diseases is not well understood and somewhat controversial depending on the disease in question, but increased GFAP staining is associated with gliosis and neuroinflammation (Jeglinski et al., 1995; Arnold et al., 1996; Iliev et al., 2001; Sawaguchi et al., 2003; Catts et al., 2014; Niranjan et al., 2014). Alphaviruses have been described to infect astrocytes and the affect is cytotoxic (Schoneboom et al., 1999). This cytotoxicity in conjunction with infection of other CNS cell types leads to neuroinflammation (Schoneboom et al., 2000). NeuN is a neuronal nuclear antigen that stains for mature neurons. Evidence for increased GFAP, potentially indicating gliosis and neuroinflammation, was observed in the dentate gyrus and posterior complex of the thalamus of symptomatic mice, but not asymptomatic or control mice (**Figure 4**). The observation of increased GFAP only in the dentate gyrus, a region of the hippocampus important to memory, and posterior complex of the thalamus indicates potential alphavirus susceptibility in those regions. Alternatively, no change in the expression of NeuN was detected (**Figure 4**), indicating that there was no significant loss or gain of neurons as a result of this infection. These data indicate that astrocytes and their secreted cytokines may play a larger role in the development of sequelae than neurons, but additional studies will be required to delineate these roles.

## Conclusion

This study led to the initial development of an infection-based murine model that develops multiple neurological complications documented in human alphavirus infection. We acknowledge that our small sample size is a limitation of this study. To further support our results and strengthen the face and construct validities of the model described above, additional studies with larger sample sizes will be required. In addition to demonstrating reproducibility of the model, specifically evaluating the neurochemical defects, resulting gene and protein expression, as well as the effects of therapeutic interventions on outcomes in infected animals will allow us and others to delineate mechanisms associated with the long-term neurological sequelae of alphavirus encephalitis. This combination of neuroscience and infectious disease will be crucial to improving the quality of life for survivors of these infections and help adapt these techniques for the study of other viral encephalitis sequelae, such as Zika virus or Ebola virus, and learn how viral infections can lead to or exacerbate neurological disorders.

## Ethics Statement

All animal experiments were approved by the IACUC at UTMB.

## Author Contributions

All authors listed have made substantial, direct, and intellectual contributions to the work and approved it for publication.

## Conflict of Interest Statement

The authors declare that the research was conducted in the absence of any commercial or financial relationships that could be construed as a potential conflict of interest.
